# Human IL-12 p40 as a reporter gene for high-throughput screening of engineered mouse embryonic stem cells

**DOI:** 10.1186/1472-6750-8-52

**Published:** 2008-06-03

**Authors:** Leonardo D'Aiuto, Clinton S Robison, Margherita Gigante, Edward Nwanegbo, Benjamin Shaffer, Meena Sukhwani, Carlos A Castro, J Richard Chaillet

**Affiliations:** 1 Department of Cell Biology and Physiology, Pittsburgh Development Center, Magee-Women's Research Institute, University of Pittsburgh School of Medicine, PA 15261, USA; 2 Department of Biological Sciences and Pittsburgh NMR Center for Biomedical Research, Carnegie Mellon University, Pittsburgh, PA 15213, USA; 3 Consorzio C.A.R.S.O, Strada Prov. Valenzano-Casamassima, Km.3 70100 Valenzano, Italy; 4 Department of Surgery, Division of Infectious Diseases, University of Pittsburgh School of Medicine, Pittsburgh, PA 15219, USA; 5 Department of Microbiology and Molecular Genetics, University of Pittsburgh, Pittsburgh, PA 15213, USA; 6 Magee-Womens Research Institute and Foundation, Pittsburgh, PA, USA; 7 Transgenic Core, Magee-Womens Research Institute Transgenic and Molecular Core, Pittsburgh, PA, USA; 8 Department of Obstetrics, Gynecology, and Reproductive Sciences, University of Pittsburgh, USA

## Abstract

**Background:**

Establishing a suitable level of exogenous gene expression in mammalian cells in general, and embryonic stem (ES) cells in particular, is an important aspect of understanding pathways of cell differentiation, signal transduction and cell physiology. Despite its importance, this process remains challenging because of the poor correlation between the presence of introduced exogenous DNA and its transcription. Consequently, many transfected cells must be screened to identify those with an appropriate level of expression. To improve the screening process, we investigated the utility of the human interleukin 12 (IL-12) p40 cDNA as a reporter gene for studies of mammalian gene expression and for high-throughput screening of engineered mouse embryonic stem cells.

**Results:**

A series of expression plasmids were used to study the utility of IL-12 p40 as an accurate reporter of gene activity. These studies included a characterization of the IL-12 p40 expression system in terms of: (i) a time course of IL-12 p40 accumulation in the medium of transfected cells; (ii) the dose-response relationship between the input DNA and IL-12 p40 mRNA levels and IL-12 p40 protein secretion; (iii) the utility of IL-12 p40 as a reporter gene for analyzing the activity of *cis*-acting genetic elements; (iv) expression of the IL-12 p40 reporter protein driven by an IRES element in a bicistronic mRNA; (v) utility of IL-12 p40 as a reporter gene in a high-throughput screening strategy to identify successful transformed mouse embryonic stem cells; (vi) demonstration of pluripotency of IL-12 p40 expressing ES cells *in vitro *and *in vivo*; and (vii) germline transmission of the IL-12 p40 reporter gene.

**Conclusion:**

IL-12 p40 showed several advantages as a reporter gene in terms of sensitivity and ease of the detection procedure. The IL-12 p40 assay was rapid and simple, in as much as the reporter protein secreted from the transfected cells was accurately measured by ELISA using a small aliquot of the culture medium. Remarkably, expression of Il-12 p40 does not affect the pluripotency of mouse ES cells. To our knowledge, human IL-12 p40 is the first secreted reporter protein suitable for high-throughput screening of mouse ES cells. In comparison to other secreted reporters, such as the widely used alkaline phosphatase (SEAP) reporter, the IL-12 p40 reporter system offers other real advantages.

## Background

There are many different reasons for expressing exogenous DNA sequences in mammalian cells. These reasons range from the necessity of gene therapy clinical trials or functional genomics analysis to simply the desire to analyze *cis*-acting genetic regulatory elements. In the latter instances, the introduced DNA constructs are usually referred to as reporter constructs because the genes expressed from these constructs report the presence of the introduced sequences. Regulatory genetic sequences of interest are combined with a reporter gene of choice to generate constructs in which the genetic elements control reporter expression. In most cases, the expression level of a reporter gene will correlate with the transcriptional activity of the regulatory genetic sequence of interest [1]. In addition to this largely predictable effect, there are also important, poorly understood, stochastic effects on the level of reporter expression.

In recent years, the simple notion of a reporter of sequence function has greatly expanded. Now, reporter genes are used to visually identify transformed cells, calculate the efficiency of gene delivery systems, follow the intracellular fate of a gene product, monitor recombination events [2], measure signal transduction [3], detect the interaction of two proteins in the two-hybrid system [4], or for noninvasive *in vivo *imaging of gene expression. Examples of the latter are positron emission tomography [5], magnetic resonance [6] and optical imaging systems [7].

Despite these diverse uses of reporters, a steady and high level of gene expression is frequently the common goal with all uses. To achieve this, reporters have been used whose expression directly reflects the expression level of the gene of interest. The coexpression of heterologous gene products in a single vector is usually accomplished with either two independent promoters or an internal ribosome entry site (IRES) [8] sequences placed between two cDNAs to transcribe a bicistronic mRNA from a single promoter. However, the use of heterologous promoters can cause promoter interference, i.e., transcription from one promoter suppresses transcription from one another [9]. Furthermore, a correlation between reporter expression and gene-of-interest expression is frequently lacking in these experiments. The use of IRES sequence in bicistronic constructs eliminates promoter interference problems and directly couples the expression of a reporter gene to the expression of the gene of interest [10]. In most cases, the protein expression of the IRES-dependent gene ranges between 20 and 50% of the protein expression of the 5' gene in the bicistronic message [11]. The arrangement and composition of reading frames can occasionally influence the strength of IRES-dependent translation [12].

Depending upon the application, an ideal reporter should have the following features: (i) the reporter protein should be absent from the host or otherwise easily distinguishable from endogenous proteins; (ii) it should be inert and not affect the physiology of the cell; and (iii) simple, sensitive, and inexpensive methodologies methods should be available to detect and quantify reporter expression. Currently, two types of reporter genes are commercially available and are classified as intracellular or extracellular reporter genes. Intracellular reporter genes include: Chloramphenicol acethyltransferase (CAT) [13], β-galactosidase [14], aequorin [15], green fluorescent protein (GFP) [16], firefly [17] and bacterial luciferase [18] and glucuronidase [19]. Extracellular reporter gene products are secreted into the medium culture. These include: human growth hormone (HGH) [20] and secreted alkaline phosphatase (SEAP) [21].

In this paper, we describe the use of the human interleukin-12 (IL-12) p40 as a secreted reporter protein. IL-12 is a heterodimeric cytokine of 70 kDa composed of two chains, a heavy chain of 40 kDa (p40) and a light chain of 35 kDa (p35) [22]. Intact IL-12 is necessary for the T cell-independent induction of interferon (IFN-γ)-gamma, for the development of a Th1 response, and for the activation of differentiated T lymphocytes of both CD4+ and CD8+ phenotypes [23]. The p40 subunit is thought to be primarily involved in receptor binding while p35 is critical for signal transduction [24]. IL-12 p40 by itself does not have any IL-12 bioactivity; it is expressed only by macrophage and dendritic cells in response to antigenic stimulation, and its expression is easily detectable by ELISA. Because of this assembly of nearly ideal features, we explored the potential use of the IL-12 p40 as reporter gene to measure the activity of *cis*-genetic elements, and in high-throughput screenings of engineered mammalian cells. Our results show that the Il-12 p40 is a very useful reporter gene. The protein assay is easy, inexpensive, and it is adaptable to applications that require sensitive quantitation or simply 'yes' or 'no' determination of gene expression. In particular, the Il-12 p40 has several features that make it a particularly attractive reporter gene when compared to the commonly used SEAP.

## Results

### Time course of IL-12 p40 accumulation

**Figure 1:**
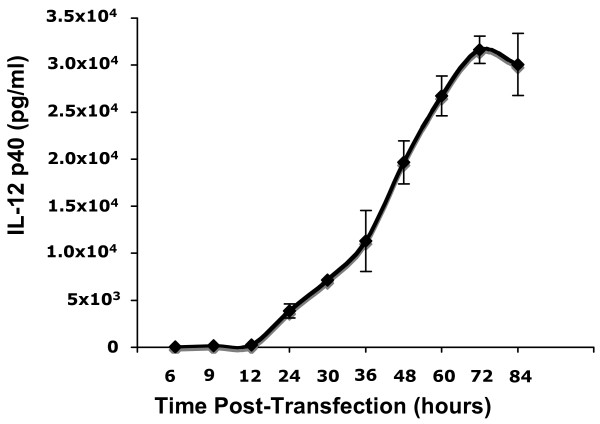
**Time course of IL-12 p40 activity changes in transfected cells**. COS-7 and BHK-21 cell lines were transfetced with 100 ng each of PGK-p40 as described in MATERIALS AND METHODS. Supernatant media was collected 6, 9, 12, 24, 48 and 72 hours after transfecion and the IL-12 expression was measured for each time point by ELISA. Values of IL-12 p40 expression shown are the average of three plates transfected in parallel.

BHK-21 cell were transfected with PGK-p40 plasmid (see Methods), and the time course for the expression of IL-12 p40 was determined (Figure 1). IL-12 p40 was first detected in the medium of transfected cells 12 hours after transfection. The maximum IL-12 p40 concentration was observed 72 hours after transfection.

### Measurement of IL-12 p40 secreted protein and mRNA levels

**Figure 2:**
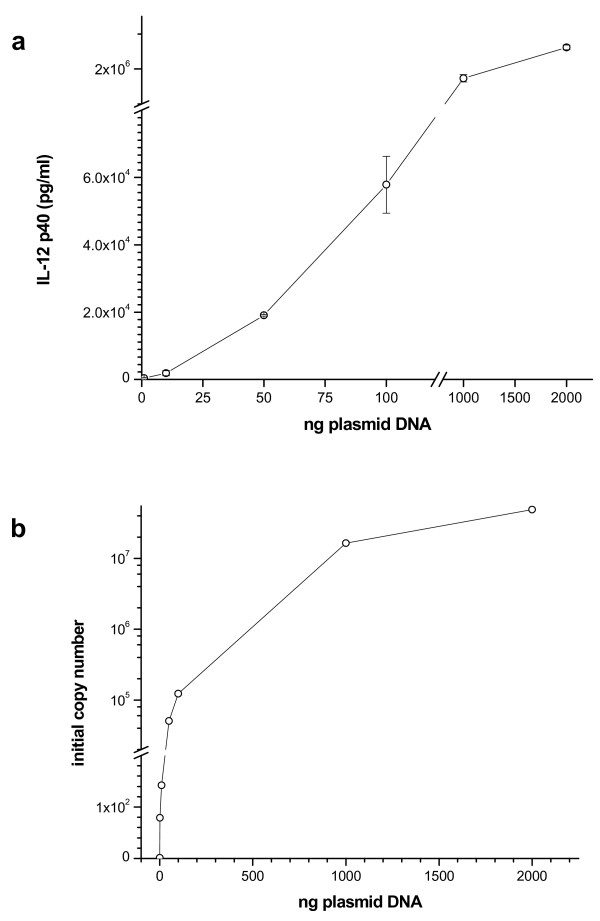
**Relationship between the IL-12 p40 activity and the amount of the transfected PGK-p40**. **a**) BHK-21 cells plated in 24-well plates at the density of 10^5^/well were transfected with the indicated amount of PGK-p40 plasmid. **b**) initial copy number of cDNA transcribed (+RT) from cell samples transfected with various amounts of the p40-encoding plasmid. The initial copy numbers were determined by QPCR as described in the methods section by using a FAM-labeled primer set specific for IL-12b (Certified Lux™ primer set 310H-01, Invitrogen). Samples incubated in the absence of RT (-RT) produced no significant amplification and were omitted from the calculations. The Ct (dRn) values of the samples were plotted against a standard curve of known copy numbers of p40-encoding plasmid run in parallel (RSq = 0.982) to obtain the initial copy number values.

10^5 ^BHK-21 cells were transfected with different amounts of PGK-p40 plasmid. On the basis of the result of the time course analysis (Figure 1), media were collected after 18 hours and assayed by ELISA. At this time the cells were harvested for the preparation of the cytoplasmic RNA. Figure 2a shows the relationship between IL-12 p40 concentration in the medium and the amount of PGK-p40 used for transfection of BHK-21 cells. The amount of IL-12 p40 secreted after transfection increased with the amount of the plasmid transfected up to 1 μg DNA when it reached a plateau.

To correlate the p40 levels detected by ELISA with transcription levels, QRT-PCR was performed on RNA extracted from the transfected cells. The number of IL-12 p40 transcripts were roughly proportional to the amount of transfected PGK-p40 plasmid (figure 2b). These results show that the amount of IL-12 p40 secreted into the medium is directly proportional to the amount of the mRNA present in the cells.

### Evaluation of relative promoter strength using EGFP and IL-12 p40 as reporter genes

**Figure 3:**
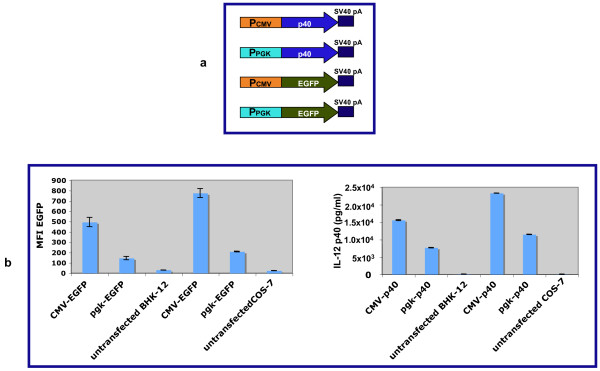
**Analysis of the CMV and PGK promoters in BHK-21, and COS-7 cells**. **a**) Structures of the CMV-p40, PGK-p40, CMV-EGFP, and PGK-EGFP constructs. **b**) Transient EGFP and Il-12 p40 expression in transfected SHK and COS-7. EGFP mean fluorescence intensity (MFI) was measured by flow flow cytometry, and IL-12 p40 expression was assayed by ELISA. Values of EGFP and IL-12 p40 expression shown are the average of three plates transfected in parallel.

The functional activity of the PGK and CMV promoters were compared by transfecting CMV-p40 and PGK-p40 constructs into BHK-21 and COS-7 cells. To measure the transfection efficiencies and compare extracellular IL-12 p40 concentration to the intracellular EGFP reporter in these experiments, the CMV-p40 and PGK-p40 constructs were cotransfected with CMV-EGFP and PGK-EGFP, respectively. EGFP and IL-12 p40 activities were measured after cotransfection of equal amounts of CMV-EGFP and CMV-p40, or PGK-EGFP and PGK-p40. The IL-12 p40 expression was assayed by ELISA sixteen hours after transfection. Data obtained using 10 μl of media are shown (figure 3). Transfected cells were examined for EGFP expression using the FACScan flow cytometer. The EGFP mean fluorescence intensity (MFI) was determined for each experiment and the results compared to the amounts of the IL-12 p40 secreted into the medium. Figure 3 shows that the difference between the strengths of the CMV and PGK promoters measured using EGFP and IL-12 p40 were similar. IL-12 p40 was not detected in untransfected control cultures.

### IL-12 p40 as a reporter gene for bicistronic plasmids

**Figure 4:**
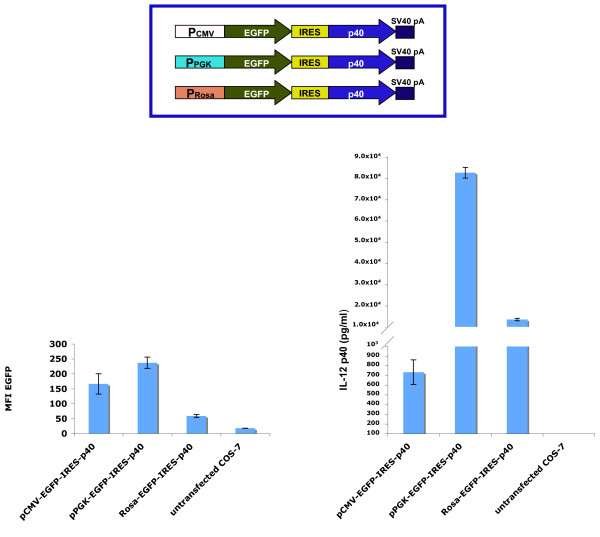
**Comparison of EGFP and IL-12 expression in the COS-7 cells transfected with pCMV-EGFP-IRES-p40, pPGK-EGFP-IRES-p40, and pRosa-EGFP-IRES-p40**. a) schematic diagram of pCMV-EGFP-IRES-p40, pPGK-EGFP-IRES-p40, and pRosa-EGFP-IRES-p40 plasmids. B) The expression of EGFP and IL-12-p40 from the indicated plasmid were determined. EGFP mean fluorescence intensity (MFI) was measured by flow cytometry, and IL-12 p40 expression was assayed by ELISA. Values of EGFP and IL-12 p40 expression shown are the average of three plates transfected in parallel.

The IRES-based bicistronic vectors are powerful tools in molecular and cellular biology, because the expression of a reporter gene from the bicistronic message correlates well with the expression of the second gene [10]. We examined the expression of the IL-12 p40 reporter protein driven by the encephalomyocarditis (EMCV) IRES element in bicistronic mRNAs. The importance of this analysis is dictated by the observation that the expression levels of various cytokines and reporter genes in the second cistron of bicistronic mRNAs is very low. This observation is interpreted in terms of negative effect that some sequences can exert on IRES-mediated translation, regardless of the nature and sequence of the IRES elements [12]. We analyzed the expression of the IL-12 p40 in the second cistron of bicistronic mRNAs whose transcription is driven by different promoters. The PGK and Rosa26 promoters were cloned into pΔEnh-IRES-p40 to generate pPGK-IRES-p40 and pRosa-IRES-p40, respectively. To measure the transfection efficiency of the vectors and compare the IL-12 p40 expression to a well-established reporter protein, the EGFP coding sequence was inserted upstream to the IRES element of the pCMV-IRES-p40, pPGK-IRES-p40 and pRosa-IRES-p40, and the resulting plasmids were named pCMV-EGFP-IRES-p40 pPGK-EGFP-IRES-p40 and pRosa-EGFP-IRES-p40 (figure 4). The first cistron, encoding EGFP, will be translated by a cap-dependent mechanism, whereas the second cistron encoding IL-12 p40 will require translation by IRES. The above constructs were transfected into COS-7 cells, and EGFP and IL-12 p40 expression measured. IL-12 p40 levels were normalized for transfection efficiency based on percentage of EGFP positive cells. IRES-mediated translation of extracellular IL-12 p40 was proportional to the EGFP mean fluorescence intensity (EGFP MFI) of the COS-7 cells transfected with PGK-EGFP-IRES-p40 and pRosa-EGFP-IRES-p40 (figure 4). This is consistent with the concept that IL-12 p40 expression is proportional to the steady-state level of the bicistronic mRNA. IL-12 p40 expression from pCMV-EGFP-IRES-p40 was detectable but very low compared to the expression from pPGK-EGFP-IRES-p40 and pRosa-EGFP-IRES-p40. This is an unexpected result given the relatively high level of EGFP MFI from pCMV-EGFP-IRES-p40, and the identical EGFP-IRES-p40 structure in all three plasmids. Because the plasmids differ only in their resident promoters, we would expect a similar rate of EGFP to IRES-dependent IL-12 p40 translation in all three plasmids. This unexpected low IL-12 p40 expression from COS-7 cells transfected with CMV-EGFP-IRES-p40 indicates that the CMV promoter likely exerts a negative effect on IRES-mediated translation. To our knowledge, this is the first evidence of inhibitory activity of a promoter element on IRES-mediated translation.

### Use of IL-12 p40 as a reporter gene in a high-throughput screening strategy to identify successful transformed cells

**Figure 5:**
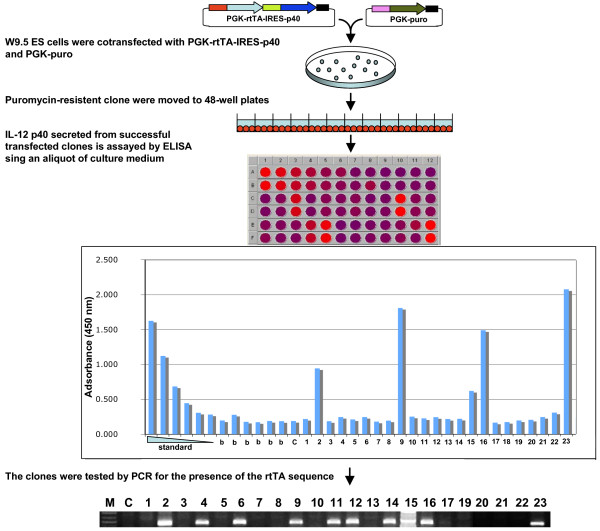
**Example of the IL-12 based screening**. ES cells successfully transfected with a bicistronic construct expressing IL-12 40, secrete IL-12 p40 into the culture medium if the espression of this reporter protein is not repressed. ES colonies expressing IL-12 p40 are identified by ELISA using a small aliquot of culture medium, and subsequently tested by PCR. All standards, samples and controls were run in duplicate and mean adsorbance values calculated. Standard ranges between 2000–62 pg/ml; c = untransfected; b = blank.

The ease of accurately measuring IL-12 p40 secreted from transfected ES cells in culture prompted us to use the IL-12 p40 to develop a high-throughput screening strategy to identify successfully modified mouse embryonic stem (ES) cells. We chose to use the IL-12 p40 as a reporter gene to rapidly identify transformed mouse embryonic stem cells expressing the reverse tetracycline controlled transactivator (rtTA) [25]. W9.5 mouse embryonic stem cells were electroporated with PGK-rtTA-IRES-p40 and PGK-puromycin-resistance constructs. After puromycin selection for 8–10 days, fifty clones were transferred into individual wells of 48-well plates. After 4 days, the IL-12 p40 concentration in the medium of transfected cells was assayed by ELISA using 100 μl of the culture medium from 23 wells containing similar cell densities. All samples were run in duplicate. ELISA assay showed relatively high IL-12 p40 expression in five puromycin-resistant clones (22%). Out of 23 clones, 10 (43%) contained rtTA sequence, determined by PCR assay (figure 5). Therefore, 50% of rtTA-containing clones expressed IL-12 p40. Thus, the ELISA procedure nicely identified those expressing cells out of those electroporated with PGK-rtTA-IRES-p40.

### Analysis of the pluripotency of mouse ES cells expressing the human IL-12 p40

**Figure 6:**
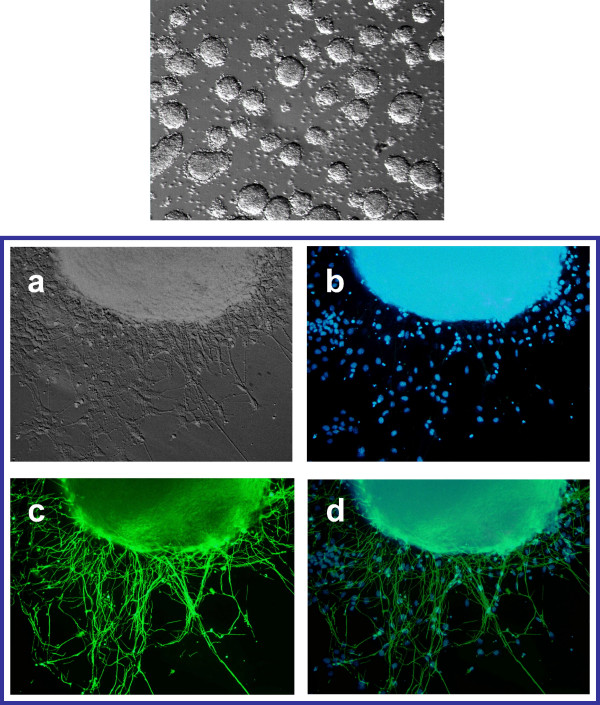
**In vitro differentiation of mouse ES cells expressing IL-12 p40. *Top*. embryoid bodies derived from mouse embryonic stem cells overexpressing IL-12 p40**. *Bottom*. Neuronal differentiation of mouse W9.5 ES cells overexpressing IL-12 p40. a) Phase contrast image of an embryoid body differentiating into a defined neurona lineage. b) DAPI counterstaining. c) neuron specific class III β-tubulin. d) Overlay.

We investigated whether IL-12 p40 expression compromises the pluripotent properties of the ES cells. One of the clones expressing IL-12 p40 was cultured in suspension in EB medium (see Methods). These ES cells expressing IL-12 p40 are able to form embryoid bodies (EBs) in 3–5 days (figure 6). Following attachment and growth of these EBs in N2 medium for 5–7 days, cells with neuronal morphology began to appear within less than 2 days. After 5 days, most of the neurons were tubulin-positive, indicating that the expression of the IL-12 p40 does not interfere with the ability of ES cell to differentiate in vitro (figure 6).

**Figure 7:**
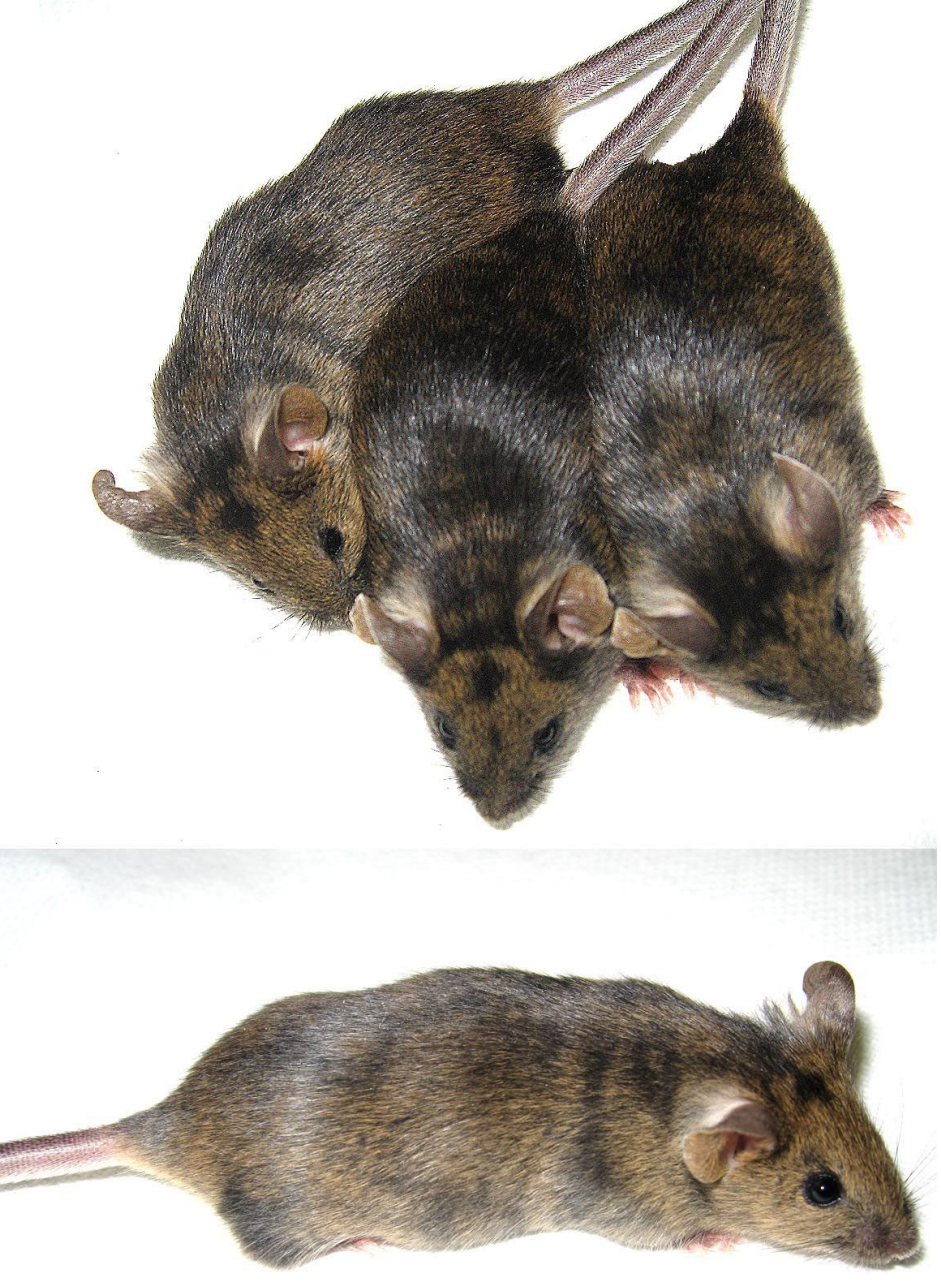
**Photographs of chimeric mice generated from the same p40-expressing ES clone (clone pgk164-p40-19)**. The mice were two months of age at the time of the photograph.

To determine the in vivo developmental potential of p40-expressing ES cells, we used one high-expressing clone (pgk164-p40-19 also expressing a bicistronic mRNA encoding a version of the mouse CIITA gene in addition to p40) to generate two female and four male chimeric mice. This ES cell clone expressed a p40 protein at a level comparable to p40-expressing ES clone 9 in figure 5 (data not shown). Four chimeras are shown in figure 7. From the extensive amount of agouti fur in these mice, we estimated their overall ES cell contribution to be greater than 80%. This p40-expressing ES clone likely contributes to the developmental of all tissues of the mouse to a similar level (80%) as seen in the fur. Contribution to the germ lineage of one of the chimeras was confirmed by demonstrating the transmission of the CIITA/p40 bicistronic construct to his offspring (see Additional File 1).

**Figure 8:**
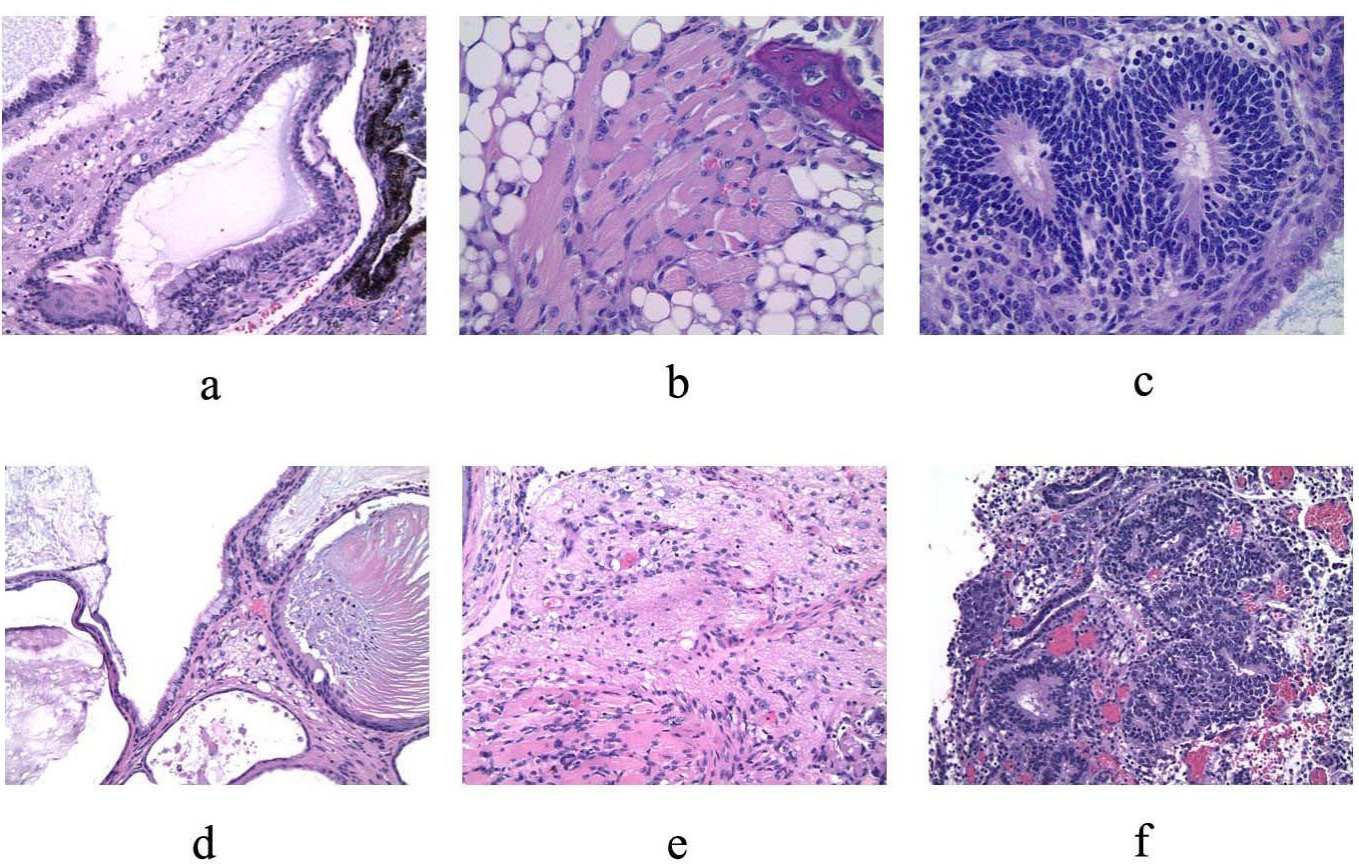
**Histologic sections of teratomas resulting from in vivo differentiation of mouse ES lines expressing IL-12 p40**. Tissues from all three primary cell lineages (endo-, meso-, and ectoderm) were formed in individual teratomas. **a-c**. Sections from a single teratoma derived from an R1 cell line expressing a high level of IL-12 p40 from pPGK-cIITA-IRES-p40. **d-e**. Sections from a single teratoma derived from a W9.5 cell line expressing a high level of IL-12 p40 from pPGK-rtTA-IRES-p40. **a, d**. ciliated epithelium of endodermal origin. **b, e**. muscle of mesodermal origin. **c, f**. neuroectoderm.

Teratomas were generated from p40-expressing ES cells to further demonstrate their pluripotency. Teratomas were successfully produced from all lines tested (6 lines total, 3 of which expressed bicistronic rtTA/p40 mRNA and p40 protein in W9.5 ES cells, and 3 of which expressed bicistronic CIITA/p40 mRNA and p40 protein in R1 ES cells). Tissues derived from all three primary cell lineages (endo-, meso- and ectoderm) were seen in sections of teratomas from each of the 6 lines examined (figure 8). We conclude from the aforementioned analyses that p40-expressing ES cells are developmentally pluripotent, and that the expression of p40 protein has no adverse effect on the inherent pluripotency of mouse ES cells.

## Discussion

Standard methods to generate stable cell lines requires transfecting a host cell line with two expression cassettes: one expressing the gene of interest and the other expressing the antibiotic resistance marker, which can be placed together on the same vector or on two distinct vectors. The level of gene expression using such screening strategies cannot be predicted. Clones expressing the gene of interest at high levels can be identified if the cells are transfected with a bicistronic construct containing the gene of interest and a reporter gene encoding for a visual or secreted reporter protein. We used the IL-12 p40 reporter gene in gene delivery systems as a rapid and cost-effective high-throughput screening strategy to identify successfully transformed cells (figure 5). The Il-12 p40 secreted from the transfected cells can be assayed by ELISA using a small aliquot of the culture medium. As little as 3.9 pg/ml of IL-12 p40 can be detected [26]. Since there is no need to prepare cell lysate to measure IL-12 p40 expression, the transfected cells can be utilized for further investigations. The absence of a cell lysis step decreases assay variability. We found that IL-12 p40 has several features that make it a particularly attractive reporter gene when compared to another secreted protein which is being used widely, that is secreted alkaline phosphatase (SEAP): i) the IL-12 is produced by macrophages and dendritic cells only in response to antigenic stimulation. The restricted nature of expression of the IL-12 permits the use of the p40 as a reporter protein in most mammalian cells. In contrast, the SEAP is not appropriate for cells derived from testes, cervix, and lung, as these have low levels of placental-type alkaline phospatase [27]; ii) the cost of the Il-12 p40 ELISA kit is 3–5 times less than the SEAP assay kit; iii) high-throughput screening strategies based on the use of the IL-12 p40 as reporter gene do not require special equipments, such as luminometer or spectrophotometer. Interpretation of the IL-12 p40 ELISA assay can be simple and straightforward; comparison of the expression levels among different clones can be made by visual inspection, based on the color developed in the positive wells of the microplates during the last two steps of the assay; iv) expression of the human IL-12 p40 does not compromise the developmental potential of mouse ES cells.

Recently, two secreted reporter proteins have been described: secreted GFP (secGFP) [28] and Gaussia luciferase (hGLuc) [29]. However, lack of information does not allow us to compare these novel reporters with IL-12 p40. secGFP has been tested in plants but not in mammalian cells, and hGLuc expression has not been analyzed in bicistronic mRNAs. The latter point is crucial when a reporter gene is used to indirectly measure the expression of a gene of interest. IRES-driven translation efficiency of the second cistron in bicistronic mRNAs is influenced by the composition of both reading frames [12]. We therefore analyzed the IRES-dependent IL-12 p40 expression in bicistronic mRNAs carrying three different upstream cistrons: EGFP (figure 4), rtTA (figure 5) and dominant negative CIITA (data not shown). In all cases, the IL-12 p40 expression was efficient and independent of the composition of the first cistron.

IL-12 p40 has advantages over the chloramphenicol acetyltransferase (CAT) [30], which is one of the most frequently used reporter proteins. Recently, the common radioactive CAT assay has been replaced by an ELISA assay [31]. As CAT is not a secreted protein, preparation of cell lysate is needed before measuring the CAT protein by ELISA. Furthermore, the sensitivity of the CAT ELISA is approximately 50 pg/ml [32] that is 15-fold less sensitive than IL-12 p40. Furthermore, IL-12 p40 offers a distinct advantage over the most widely used screening strategies that rely on the use of bicistronic vectors carrying a selection marker (e.g. neo^R^) after the IRES element. This type of bicistronic vectors can become silent under non-selective conditions. Although cell clones with homogenous transgene expression can be kept under selective conditions over a long period, this desirable phenotype can be progressively lost upon withdrawal of selective pressure [33].

## Conclusion

**Figure 9:**
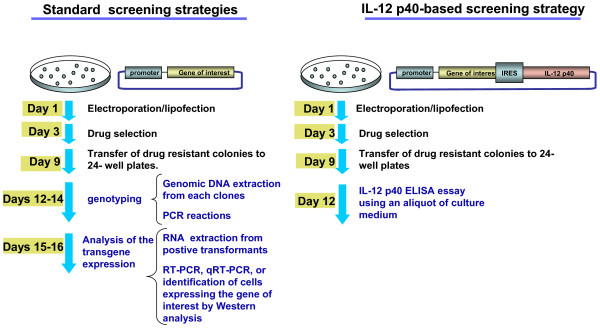
Standard screening strategies of transfected embryonic Stem (ES) cells versus screening using IL-12 p40 as reporter gene.

IL-12 p40 is a sensitive reporter gene to measure molecular genetic events in most of the eukaryotic cell types. The protein assay is adaptable to applications that require sensitive quantification or simply 'yes' or 'no' determination of gene expression. Like most commercially available reporter technologies, IL-12 p40 is not versatile enough to be considered an "all purpose" reporter gene. For instance, like luciferase and SEAP, Il-12 p40 cannot be used for fluorescence-activated cell sorting, or *in vivo *imaging gene expression analysis. However, the IL-12 p40 reporter gene can be used as a rapid and cost-effective strategy in high-throughput screening to identify successful transformed cells. Our study shows that Il-12 p40-based screening of transfected mouse ES cells offers improvement in cost, throughput, cell culture effort (figure 9). High throughput screening of mouse ES cells can be performed in any laboratory without the use of expensive automation systems.

## Methods

### Cell cultures and transfections

The BHK-21 hamster cell line (ATCC CCl-10) and the COS-7 cell line (ATCC CRL-1651) were cultured with Dulbecco's modified eagle medium (DMEM) supplemented with 10% FCS, 100 μg Streptomycin/ml, 100 U Penicillin/ml (all reagents from Chemicon/Millipore). W9.5 mouse embryonic stem (ES) cells were grown on γ-irradiated embryonic fibroblast feeder cells in DMEM supplemented with 15% fetal calf serum, 100 μg Streptomycin/ml, 100 U Penicillin/ml, and 100 U LIF (Chemicon/Millipore). Cultures were maintained in a humidified chamber in a 5% CO_2_/air mixture at 37°C. DNA transfection into BHK-21 and COS-7 cells were carried out by lipofectamine 2000 (Invitrogen). For promoter strength analysis (Figure 1) cells in exponential growth were seeded (7.5 × 10^4^) into 24-well plates the day before transfection. Cells were cotransfected with equal amount (25 ng) of pEGFP-N1 and pPGK-p40, or with pPGK-EGFP together with pPGK-p40. The procedure used for the time course of IL-12 p40 accumulation analysis (figure 1) and transfections with bicistronic constructs was similar to that described above, and 10 ng of pPGK-p40 and 150 ng of pPGK-EGFP-IRES-p40, pRosa-EGFP-IRES-p40 plasmid per well were used. All transient transfections were carried out three times. EGFP expression was analyzed using the FACScan flow cytometer and CellQuest software (BD; Mountain View; California); for each sample, 10,000 events were acquired. Non-transfected BHK-21 and COS-7 cells were used as negative controls whereas cells transfected with EGFP plasmid served as positive controls.

For electroporation in ES cells, 5 × 10^6 ^cells in PBS buffer were transfected with 20 μg linearized pPGK-rtTA-IRES-p40 (into W9.5 ES cells) or 20 μg linearized pPGK-CIITA-IRES-p40 (into R1 ES cells) plus 2 μg linearized pPGK-puro (gift from Dr. Deborah Chapman) constructs using the BioRad Gene Pulser II (200 V, 500 μF). Puromycin resistant clones were picked after 8–10 days of puromycin (1 μg/ml) selection and propagated using the same medium.

### Isolation of the human IL-12 p40 cDNAs

IL-12 p40 cDNA was isolated from a human spleen cDNA library by nested PCR using the following primers: p40-1 (ctgtttcagggccattggactctccgtcct) and p40-2 (atcttccacttttcctccaaattttcatc) for the first PCR, and p40-3 (ttatctagatccaccatgtgtcaccagcagttggtcatctcttgg) and p40-4 (atcgcggccgaataactgcagggcacagatgcccattcgctc) for the second PCR. PCR reactions were subjected to 25 cycles at 98°C for 30 s, 55°C for 30 s, and 72°C for 20 s, followed by a 10 min extension at 72°C using the Invitrogen Taq DNA polymerase.

The PCR product was purified by electrophoresis in an agarose gel and ligated into pCR2.1 TA cloning vector (Invitrogen, Carlsbad, CA) and subsequently transformed into One Shot Top10F' competent cells according to the procedures provided with the TA cloning kit. Cloned fragments were sequenced using M13 forward and M13 reverse primers to verify the integrity of the Il-12 p40 sequence. One of the plasmids, pCRp40-3 was chosen to generate the following constructs. The IL-12 p40 cDNA was excised from pCRp40-3 by digestion with *Spe*I and *Not*I, and inserted into the *Xba*I and Not I sites of the pIRES vector (Clontech) to generate **pCMV-IRES-p40 **plasmid. The CMV enhancer-promoter was deleted by digestion with *Bgl*II and Nhe I, followed by fill-in and re-circularization by ligation, and the resulting construct was designated ΔEnh-IRESp40. This plasmid was the founder for the mono- and bicistronic plasmids described below. Our plasmid names reflect the order of the mammalian cell promoter and reporter genes.

#### pRosa-EGFP-IRES-p40

The mouse Rosa26 promoter was excised from PBII-Rosa-M2/HGHpA construct (gift from Dr. Prabir Ray) with *Bam*HI and *Cla*I, blunted with Klenow polymerase and ligated into the ΔEnh-IRESp40 vector that was linearized with *Nhe*I followed by blunting (pRosa-IRES-p40 vector). The EGFP coding sequence was excised from the pCR-EGFP plasmid (D'Aiuto L., unpublished) by *Eco*RI digestion, blunted and cloned into pRosa-IRES-p40 vector which was linearized with Mlu I and blunted. The resulting vector was named pRosa-EGFP-IRES-p40.

#### pPGK-EGFP-IRES-p40

This construct was generated similarly to ROSA-EGFP-IRES-p40. The 520 bp *Eco*RI-*Taq*I mouse Pgk-1 promoter was PCR amplified from mouse genomic DNA, cloned into *Eco*RI-*Pst*I sites of pBluescript. Pgk-1 promoter was excised from resulting construct with *Eco*RI and *Bam*HI and blunted.

#### pPGK-M2-IRES-p40

The rtTA-M2 gene was excised from PBII-Rosa-M2/HGHpA construct (gift from Dr. Prabir Ray) by digestion with Bam HI and Cla I. The resulting fragment was blunted and ligated into pPGK-IRES-p40 linearized with *Bam*HI and blunted.

#### pPGK-CIITA-IRES-p40

A cDNA encoding the human CIITA gene was ligated into pPGK-IRES-p40.

#### pCMV-p40 and pPGK-p40

These plasmids were derived from pCMV-EGFP-IRES-p40 and pPGK-EGFP-IRES-p40 by removing the IRES sequecnce and p40 cDNA through *Nhe*I-*Xma*I and *Ava*I restriction digestions, respectively.

#### pPGK-EGFP

The PGK promoter restricted by *Eco*RI and *Bam*HI sites was cloned into pEGFP-N1 plasmid.

#### QRT-PCR

RNA was extracted from BHK-21 cells transfected with PGK-p40 plasmid using an RNeasy kit (Qiagen) and cDNA first strand synthesis wascarried out using enhanced AMV reverse transcriptase (eAMV RT, Sigma) and oligo-dT priming according to manufacturer's recommendations. QPCR of samples that were either incubated with or without eAMV RT were quantified using a Stratagene MX3000 QPCR machine. Twenty-five microliter PCR reactions were performed using a FAM-labeled D-Luxtm primer set specific for human p40 transcript (Invitrogen) run in Strategene 2-step QPCR master mix with ROX reference dye and using 50 cycles (melt 95°C, 3 s; anneal 60°C, 10 s; extend 72°C, 30 s). To quantify transcript levels, a standard curve was run in parallel reactions and employed a serial dilution set of p40 encoding template DNAs.

### ELISA

Il-12 p40 secretion from electroporated BHK-21, COS-7 and ES cells was detected by IL-12 p40 ELISA kit (Biolegend).

### Neuronal differentiation

To promote embryoid body formation, ES cells expressing IL-12 p40 were plated at 5 × 10^4 ^ES cells/well in non-adherent 6-well plate in EB medium (DMEM containing 5% Knockout Serum Replacement, nonessential amino acids, β-mercaptoethanol, sodium pyruvate and glutamine) and grown for seven days. Medium was changed every two days. On day 7 the embryoid bodies were collected and resuspended in N2 medium and transferred to an adherent 6-well plate coated with a 0.2% gelatin. The N2 medium was based on DMEM/F12, N-2 Supplement (Invitrogen), mouse laminin I (R&D sytems), nonessential amino acids, glutamine, β-mercaptoethanol, and bFGF (Invitrogen). The N2 medium was changed every two days for 5–7 days.

### Immunocytochemistry

Cells were fixed with 4% paraformaldehyde (PFA) for 10 min at room temperature, washed in PBS and blocked for 1 h in blocking buffer (10% goat serum in PBS). Samples were incubated with Beta-Tubulin III (clone Tuj1) monoclonal antibody (Covance) for 1 hour, washed in PBS and incubated with Alexa 488 Goat-Anti mouse IgG (H+L) (Invitrogen), and counterstained with DAPI.

### Production of chimeric mice

Chimeric mice were produced by injecting 15–20 cells from CIITA/p40 expressing ES clone pkg164-p40-19 derived from R1 ES cells into blastocysts of the inbred C57B1/6J strain, followed by transfer of the injected blastocysts into the uterine horns of pseudopregnant female mice. All experiments on animals conformed to the policies of the University of Pittsburgh Institutional Care & Use Committee.

### Generation and analysis of ES-derived teratomas

Cell suspensions were introduced into the testes of immune deficient SCID mice by modified efferent duct injection [34]. The injection pipette was advanced along the efferent duct and through the rete testes into the interstitial space where cells were injected using an Eppendorf Femtojet pressure injector. Recipient mice were routinely evaluated for palpable tumors, which typically developed between two and four months after injection. H&E stained sections of tumors were analyzed.

## Authors' contributions

LD'A conceived of the study, coordinated it, partecipated in its experimental part (screening of human spleen cDNA library, clonings, transfections). CR performed QRT-PCR. MG carried out ELISA assays. EN performed EGFP expression analysis. BS designed and constructed plasmids and JRC participated in work coordination and provided critical inputs during manuscript preparation. MS generated the teratomas and CC characterized the teratomas. All authors read and approved the final manuscript.

## Supplementary Material

Additional file 1 Germline transmission of the CIITA/p40 bicistronic construct. Germline transmission was obtained after injection of embryonic stem cells expressing cIITA and IL-12 p40 into C57BL/6 blastocysts. Then, mice were intercrossed. Litters were genotyped by PCR to investigate the germline transmission of cIITA (lanes 2–8) and IL-12 p40 (lanes 11–17). M = 1 kb ladder, N = negative control (R1 genomic DNA), P = positive control (pgk-164-p40 construct). Click here for file
