# Supplementary figures and images for: Human IL-12 p40 as a reporter gene for high-throughput screening of engineered mouse embryonic stem cells
Source: BMC Biotechnol. 2008 Jun 3;8:52. doi: 10.1186/1472-6750-8-52 (PMC2442052; doi:10.1186/1472-6750-8-52)

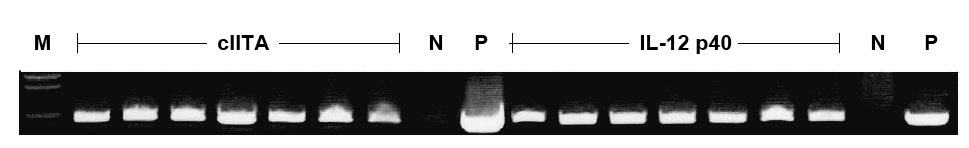

Supplement: Additional file 1 — Germline transmission of the CIITA/p40 bicistronic construct. Germline transmission was obtained after injection of embryonic stem cells expressing cIITA and IL-12 p40 into C57BL/6 blastocysts. Then, mice were intercrossed. Litters were genotyped by PCR to investigate the germline transmission of cIITA (lanes 2–8) and IL-12 p40 (lanes 11–17). M = 1 kb ladder, N = negative control (R1 genomic DNA), P = positive control (pgk-164-p40 construct). [file 1472-6750-8-52-S1.jpeg]
